# Metformin-induced AMPK activation promotes cisplatin resistance through PINK1/Parkin dependent mitophagy in gastric cancer

**DOI:** 10.3389/fonc.2022.956190

**Published:** 2022-10-25

**Authors:** Yi-Yi Xiao, Jin-Xing Xiao, Xiao-Yu Wang, Tao Wang, Xin-Hui Qu, Li-Ping Jiang, Fang-Fang Tou, Zhi-Ping Chen, Xiao-Jian Han

**Affiliations:** ^1^ Institute of Geriatrics, Jiangxi Provincial People’s Hospital, The First Affiliated Hospital of Nanchang Medical College, Nanchang, China; ^2^ Department of Pharmacology, School of Pharmaceutical Science, Nanchang University, Nanchang, China; ^3^ Department of Neurology, Jiangxi Provincial People’s Hospital, The First Affiliated Hospital of Nanchang Medical College, Nanchang, China; ^4^ Department of Oncology, Jiangxi Provincial People’s Hospital, The First Affiliated Hospital of Nanchang Medical College, Nanchang, China; ^5^ Department of Critical Care Medicine, Jiangxi Provincial People’s Hospital, The First Affiliated Hospital of Nanchang Medical College, Nanchang, China

**Keywords:** cisplatin resistance, AMPK, mitophagy, gastric cancer, metformin

## Abstract

Gastric cancer (GC) is one of the most common tumors worldwide, and cisplatin is a standard chemotherapeutic reagent for GC treatment. However, chemoresistance is an inherent challenge which limits its application and effectiveness in clinic. This study aims to investigate the mechanism of metformin-induced cisplatin resistance in GC. Intriguingly, the upregulation of mitophagy markers, mitochondrial fission, autophagy and mitophagosome were observed in SGC-7901/DDP cells compared to those in the SGC-7901 cells. Treatment with metformin significantly increased mitochondrial fission and mitophagy in both AGS and SGC-7901 cells, resulting in decreased ATP production, which unexpectedly protected GC cells against the cytotoxicity of cisplatin. In contrast, application of Chloroquine and 3-methyladenine, two inhibitors of autophagy, significantly alleviated the protective effect of metformin on SGC-7901 and AGS cells against cytotoxicity of cisplatin. Moreover, metformin also stimulated the phosphorylation of AMPK (Thr172) and increased the expression of mitophagy markers including Parkin and PINK1 in the AMPK signaling-dependent manner. Consistently, the cell viability and cell apoptosis assay showed that metformin-induced cisplatin resistance was prevented by knockdown of AMPKα1. Taken together, all data in this study indicate that metformin induced AMPK activation and PINK1/Parkin dependent mitophagy, which may contribute to the progression of cisplatin resistance in GC.

## Introduction

Gastric cancer is a fatal disease with low survival rate worldwide. It is reported that there are over one million new cases every year, and gastric cancer is the fifth largest diagnosed malignant tumor in the world (1). Unfortunately, gastric cancers usually are not sensitive to immune checkpoint inhibitor monotherapies, which makes surgery and chemotherapy the principal approaches for treatment of GC. Cisplatin (DDP) is the first-line reagents for GC chemotherapy. However, cancer cells often develop resistance in the long-term use of cisplatin, which inevitably lead to primary or acquired drug resistance, an obstacle of successful cancer therapy (2). Therefore, re-sensitizing gastric cancer cells to chemotherapy and investigating the mechanism of drug resistance is of clinical significance.

Mitophagy controls mitochondrial quality by degrading superfluous and damaged mitochondria, which helps maintaining cellular homeostasis in response to stress (3). Mitochondrial fission is a prerequisite for mitophagy to remove damaged organelles, whereas mitochondrial fusion neutralizes mitophagy (4). In addition, mitochondrial oxidative stress, apoptotic factor and ATP generation are also related to the mitophagy (5). PTEN-induced putative kinase 1 (PINK1)/Parkin axis is a key regulator if mitophagy under cell stress (6). Normally, full-length PINK1 enters mitochondria, where it is cleaved by protease PARL for degradation. Under stressed and mitochondrial depolarization, PINK1 locates the mitochondrial outer membrane, and phosphorylates an E3 ubiquitin ligase Parkin. Activated Parkin induces ubiquitination of multiple mitochondrial outer membrane proteins, which are then degraded by autophagosomes. AMPK is recognized as a key sensor of cell nutrition and AMPK/ULK1 axis modulate Parkin, revealing close connections between AMPK and mitophagy (7). It is reported that mitophagy plays a central role in cancer progression and tumorigenesis (8, 9), but the role of mitophagy in cisplatin resistance of gastric cancer remains largely unknown.

Metformin (1,1-dimethylbiguanide hydrochloride), an effective hypoglycemic drug, is first reported as an antidiabetic drug in 1957 (10). Metformin is an antihyperglycemic drug for type 2 diabetes (T2D) due to its minimal side effects (11). Recent studies show that metformin has a promising role in cancer therapy. Metformin mediates anti-tumor effect may occur through several mechanisms, including inhibition of cancer cell proliferation (12), enhanced apoptosis (13), reduced angiogenesis (14), inhibition of EMT (15), regulating immune response (16) and targeting cancer stem cells (17). In contrast, it is also reported that metformin alleviates chemosensitivity to cisplatin in different cancer cells (18–20). However, the role of mitophagy in metformin-induced cisplatin-resistance in GC and its underlying mechanism remains to be elucidated.

Here, we showed that mitochondrial fission and mitophagy were enhanced in cisplatin-resistant GC cells. Intriguingly, metformin increased mitochondrial fission and mitophagy and protected GC cells against the cytotoxicity of cisplatin. In the term of mechanism, metformin activated AMPK signaling and upregulated the expression of mitophagy related proteins, including PINK, Parkin and LC3B. Further, application of mitophagy inhibitors alleviated metformin-induced cisplatin resistance. These results illustrate that metformin may facilitate cisplatin resistance of GC cells by promoting mitophagy *via* AMPK-PINK1/Parkin signaling axis. Our data also warrant caution over metformin treatment in diabetes patients with GC.

## Materials and methods

### Cell culture

Human gastric cancer AGS, SGC-7901 cells and cisplatin‐resistant SGC-7901/DDP cells were purchased from the Cell Biology of Institute of Chinese Academy of Sciences (Shanghai, China). SGC-7901 and SGC-7901/DDP cells were cultured in RPMI 1640 (BI, Israel) and AGS cells were grown in DMEM/F12 medium (BI, Israel) supplemented with 1% penicillin/streptomycin (Solarbio, China) and 10% fetal bovine serum (FBS, BI, Israel) at 37°C with 5% CO_2_ in a humidified atmosphere.

### Reagents

Antibody against β-actin (#AC004,1:100000) was purchased from ABclonal Technology (Wuhan, China). Antibodies against PINK1 (#ab23707, 1:1000) and Parkin (#ab77924, 1:2000) were purchased from Abcam (Cambridge, UK). Antibodies against LC3B (#2775S, 1:1000), PINK1 (#6946S, 1:1000), AMPKα (#5832S,1:1000), Phospho-AMPKα (Thr172) (#2535S,1:1000) were purchased from Cell Signaling Technology (Danvers, MA, USA). Horseradish peroxidase (HRP)-conjugated goat anti-rabbit IgG secondary antibody (1:5000) was obtained from Proteintech (Wuhan, China). Enhanced chemiluminescence (ECL) detection kit (#KF005) was purchased from Affinity. Cisplatin and metformin were obtained from MCE (Shanghai, China). 3-Methyladenine (#S2767) was purchased from Selleck (Houston, TX, USA), and Chloroquine (#REVG1006) was obtained from Genechem (Shanghai, China).

### Cell proliferation assay

SGC-7901 and AGS cells were seeded into 96-well plates and treated with metformin for indicated time. Subsequently, Cell proliferation was measured by CCK8 kit, following the manufacturer’s instructions. Absorbance at 450 nm was recorded using an optical density reader.

### Measurement of mitochondrial DNA

Total DNA was isolated from SGC-7901 and SGC-7901DDP cells using the Universal Genomic DNA Purification Mini Spin Kit (#D0063, Beyotime, China) according to manufacturer’s instructions. The amount of mitochondrial DNA was determined by quantitative real-time PCR using following primers : mtDNA-F 5’-CGCCTCACACTCATTCTCAACC-3’ and mtDNA-R 5’-CAAGGAAGGGGTAGGCTATGTG-3’, nDNA-F 5’- AGTCCCCCACAACACTGAGA-3’ and nDNA-R 5’-AATGGCACACGACAAGGTGG-3’. Relative mitochondrial DNA levels were calculated based on the threshold cycle (Ct) as 2^−Δ(ΔCt)^.

### Mtphagy dye staining

SGC-7901 and SGC-7901/DDP cells were washed with serum-free medium twice and incubated with Mtphagy Dye working solution (#MDO1, Dojindo, Japan) at the final concentration of 100 nM for 30 min at 37°C. Cells were then washed with serum-free medium twice. Mitophagy in live cells was detected through tracking the fluorescence Mtphagy Dye under fluorescence microscope (Olympus, Tokyo, Japan).

### Cell apoptosis assay

Cell apoptosis was detected with Annexin V-FITC/PI Apoptosis kit (#556547, BD Biosciences, San Jose, CA, USA) following manufacturer’s instruction. Cells were washed with PBS and resuspended in 300 μl binding buffer. Next, cells were double-stained with 5 µl PI and 5 µl Annexin V-FITC at RT in the dark. Cells were analyzed using a flow cytometer equipped with a laser emitting at 488 nm and an optical filter FL1 (530/30 nm). Data was processed with the Follow JO software (BD Biosciences).

### siRNA transfection

SGC-7901 cells were transfected with siRNA targeting PINK1 subunit (ChemShine Biotechnology Inc, Shanghai, China), AMPK-α1 subunit (GenePharma, Shanghai, China) or scrambled siRNA (GenePharma, Shanghai, China) as a control. The targeting sequences were as follows: PRKAA1-homo-825 5’-GGGAACAUGAAUGGUUUAATT-3’(sense) and 5’-UUAAACCAUUCAUGUUCCCTT-3’ (antisense), PRKAA1-homo-477 5’-GCUUGAUGCACACAUGAAUTT-3’ (sense) and 5’-AUUCAUGUGUGCAUCAAGCTT-3’ (antisense), PRKAA1-homo-1132 5’-CCAUUCUUGGUUGCUGAAATT-3’ (sense) and 5’-UUUCAGCAACCAAGAAUGGTT-3’ (antisense). PINK1-si-1 5’-CGAAGCCAUCUUGAACACAAUdTdT-3’(sense) and 5’-AUUGUGUUCAAGAUGGCUUCGdTdT-3’ (antisense), PINK1-si-2 5’-GCCGCAAAUGUGCUUCAUCUAdTdT-3’ (sense) and 5’-UAGAUGAAGCACAUUUGCGGCdTdT-3’ (antisense), PINK1-si-3 5’-GUUCCUCGUUAUGAAGAACUAdTdT-3’ (sense) and 5’-UAGUUCUUCAUAACGAGGAACdTdT-3’ (antisense). siRNAs were transfected intosiRNAs were transfected into SGC-7901 cells with ribo-FECT CP Transfection Kit (#C10511-05, ribo, Guangzhou, China) following the manufacturer’s instructions.

### Western blot analysis

Cells were lysed in RIPA buffer [20 mM Tris (pH 7.4), 10 mM sodium orthovanadate, 20 mM sodium fluoride, 150 mM NaCl, 1 mM dithiothreitol, 0.25 mM sucrose, 500 nM okadaic acid, and 0.5% Tween 20] and whole cell extracts were put to SDS-PAGE and transferred to PVDF membranes. The membranes were blocked with 5% skim milk and then incubated with primary antibodies anti-PINK1(Abcam, UK), anti-Parkin (Abcam, UK), anti-LC3B (CST, USA), anti-AMPKα (CST, USA), anti-phospho-AMPKα-Thr172 (CST, USA), anti-Tom20 (Santa Cruz Biotechnology, United States) or anti-β-actin (ABclonal Technology, China) for 1 h at room temperature. Next, the membranes were incubated with horseradish peroxidase (HRP)-conjugated anti-mouse or anti-rabbit secondary antibodies for 1 h at room temperature. Western blot bands were detected with a chemiluminescence kit. Densitometric analysis was carried out with ImageJ software (NIH, Bethesda, MD, United States). Protein levels were normalized to β-actin.

### Measurement of intracellular ATP level

SGC-7901 and AGS cells were seeded into a 96-well plate. The ATP levels were detected with commercial ATP assay kit (Beyotime Biotechnology, China) following manufacturer’s protocol. Each well was added with mixed reagent and incubated for 15 min. ATP levels were detected with Fluorescence/Multi-Detection Microplate Reader.

### Transmission electron microscopy

SGC-7901/DDP and SGC-7901 cells were seeded at 5×10^5^ cells/culture dish (35mm) and treated with indicated reagents. Then cells were collected and fixed by 3% glutaraldehyde at 4°C followed by 1% osmium tetroxide. Next, cells were dehydrated in 30%~100% acetone, embedded with Epox 812 and cut to semithin sections. After stained with methylene bule, the ultrathin sections (60 nm~90 nm) were prepared by EM UC7 (Leica, Germany) and stained with lead citrate and uranyl acetate. The sections were detected by transmission electron microscope (JEM-1400FLASH, Electronics Co., Ltd, Japan).

### Confocal microscopy

Cells were seeded in glass-bottom culture dishes at 50-60% confluence. To label mitochondria, SGC-7901 and AGS cells were transfected with pDsRed2-Mito with Lipofectamine 2000 (Invitrogen) following manufacturer’s instructions. Then, cells were treated with 10mM metformin for 24h, Fluorescence images were acquired with confocal microscope (Olympus, FV3000D, Tokyo, Japan). Mitochondrial length was measured and analyzed in each group.

### Statistical analysis

The results were analyzed by unpaired Student’s *t*-test or one-way ANOVA. Statistical analysis was performed with GraphPad software. Data from three independent experiments are presented as mean ± SD. *p*<0.05 was considered to be statistically significant.

## Results

### Mitochondrial fission and mitophagy were increased in the cisplatin resistant SGC-7901/DDP cells

**Figure 1:**
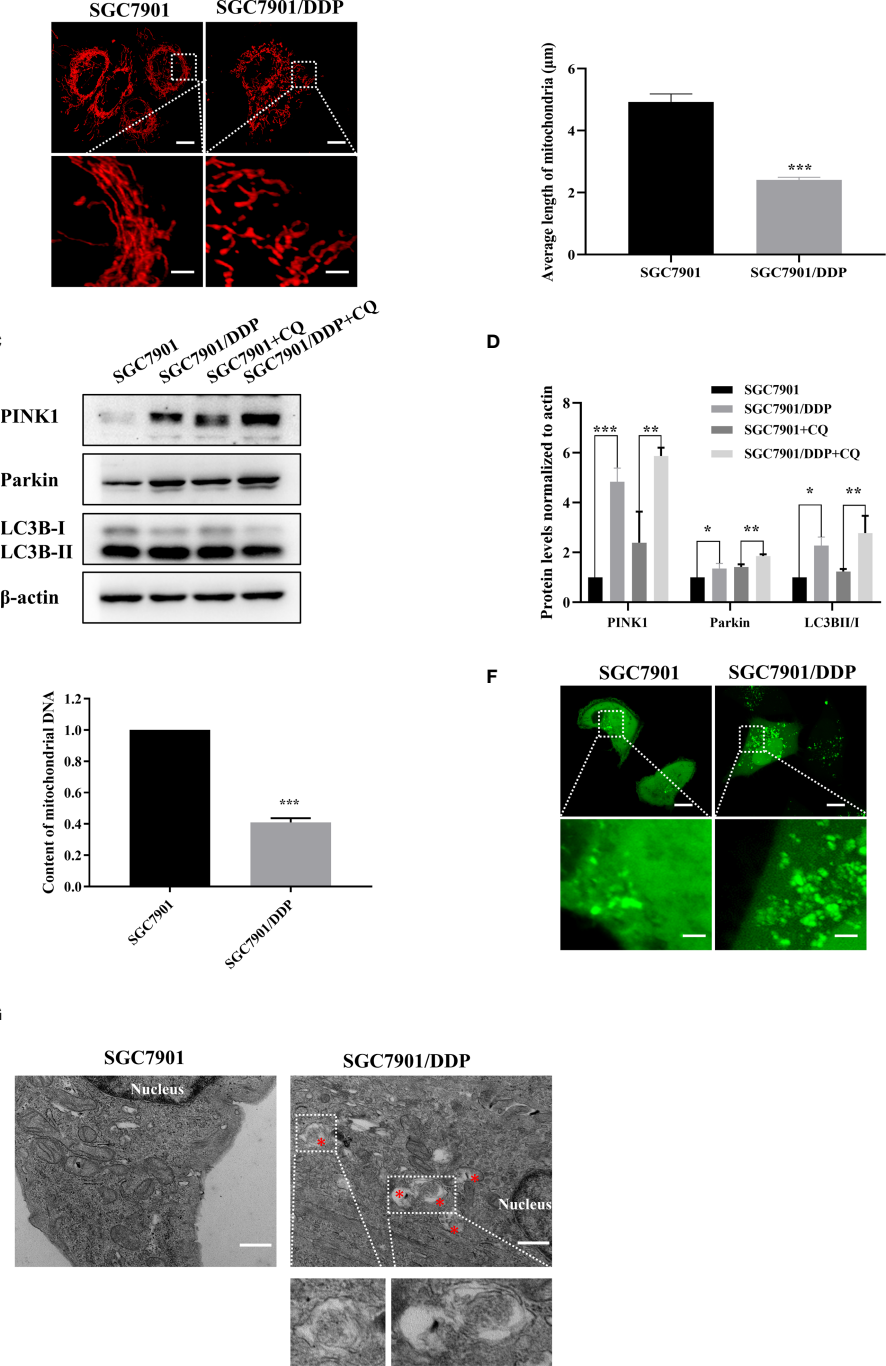
Mitochondrial fission and mitophagy were increased in the cisplatin resistant SGC-7901/DDP cells. **(A, B)** Mitochondrial morphology in SGC-7901 and SGC-7901/DDP cells. Cells were transfected with pDsRed2-Mito. Scale bar of top figures is 10 μm. Scale bar of bottom figures is 2 μm. Mitochondria length in each group was measured. **(C, D)** Expression of PINK1, Parkin and LC3B in SGC-7901 and SGC-7901/DDP cells. SGC-7901 and SGC-7901/DDP cells were treated with or without 10 µM CQ for 24 h. Whole cell lysates were collected for western blot assay. **(E)** Changes of mitochondrial DNA content. Total DNA were isolated from SGC-7901 and SGC-7901/DDP cells and the amount of mitochondrial DNA was determined by real-time PCR. **(F)** SGC7901 and SGC7901/DDP cells were transfected with pEGFP-LC3B for 24 h and then analyzed by confocal microscope. Scale bar=10 µm. Circled images at higher magnification are shown in the below panels. Scale bar= 2 µm. **(G)** SGC7901 and SGC7901/DDP cells were processed by transmission EM. Asterisk represents mitophagy vesicles. Circled images at higher magnification are shown in the below panels. Scale bar=0.5 µm. Data were presented as the means ± SD. The experiments were repeated three times independently. **p<0.01, ***p<0.001.

In this study, the cisplatin resistant SGC-7901/DDP and its parental SGC-7901 cells were used. First, the IC50 of two cell lines to cisplatin was determined by CCK8 assay. As shown in **Figure S1**, SGC-7901/DDP and SGC-7901 cells were treated with different concentrations of cisplatin and dose-response curves of cell viability were generated. IC50 values for cisplatin were calculated as 3.85 and 18.88 μg/ml in SGC-7901 and SGC-7901/DDP cells, respectively. Compared to SGC-7901 cells, the IC50 value for cisplatin against SGC-7901/DDP cells was increased 5-fold. To investigate whether mitochondrial dynamics was involved in cisplatin resistance, pDsRed2-Mito was transfected into SGC-7901 and SGC-7901/DDP cells for mitochondrial imaging. As shown in **Figures 1A, B**, mitochondria in SGC-7901 cells appeared as tubular, thread-like network, and the average mitochondrial length in SGC-7901 cells (4.9 ± 0.26 µm) was longer than that in SGC-7901/DDP cells (2.4 ± 0.09 µm). To explore the role of mitophagy in cisplatin resistance, expression of PINK, Parkin and LC3B were examined. As shown in **Figures 1C, D**, expression of PINK, Parkin and the ratio of LC3B-II/I was higher in SGC-7901/DDP cells than those in SGC-7901 cells in the presence or absence of chloroquine (CQ). Further results showed that the content of mitoDNA was decreased in SGC-7901/DDP cells compared with those in SGC-7901 cells (**Figure 1E**). The expression of TOM20, component of the TOM (translocase of outer membrane) receptor complex, was slightly down-regulated in SGC-7901/DDP cells, but it showed no significant difference between SGC-7901 and SGC-7901/DDP cells (**Figures S2A, B**). This result is consistent with previous finding that the change of mitoDNA proceeds that of mitochondrial mass (21). Then, cells were stained with Mtphagy Dye, and the fluorescence indicates the occurrence of mitophagy. As shown in **Figure S2C**, the fluorescent intensity of Mtphagy Dye is stronger in SGC-7901/DDP cells than that in the SGC-7901 cells. In accordance with western blot results, confocal imaging showed that LC3B puncta formation increased in SGC7901/DDP cells (**Figure 1F**). Finally, mitophagy in SGC-7901 and SGC-7901/DDP cells was detected by TE microscopy and the appearance of double membranes associated with mitochondria was observed in SGC-7901/DDP cells, but not in SGC-7901 cells (**Figure 1G**). These results indicate that mitochondrial fission and mitophagy are increased in the cisplatin resistant SGC-7901/DDP cells, also suggest that mitochondrial dynamics and mitophagy is involved in the cisplatin resistance in gastric cancer.

### Metformin protects GC cells from cisplatin toxicity

**Figure 2:**
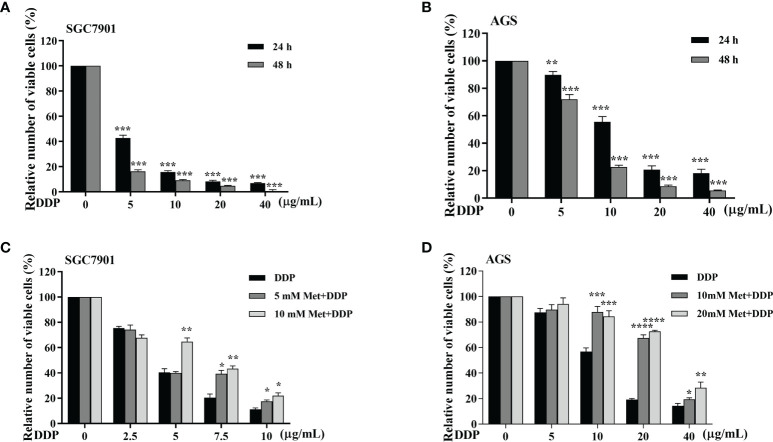
Metformin alleviates cisplatin-induced growth inhibition in GC cells. **(A, B)** SGC-7901 and AGS cells were treated with cisplatin for 24 or 48 h, followed by CCK8 assay. **(C, D)** SGC-7901 and AGS cells were pre-treated with metformin for 4 h followed with cisplatin treatment for 24 h. Cell viability was determined by CCK8 assay. Data were presented as the means ± SD. The experiments were repeated three times independently. *p<0.05, **p<0.01, ***p<0.001, ****p<0.0005.

**Figure 3:**
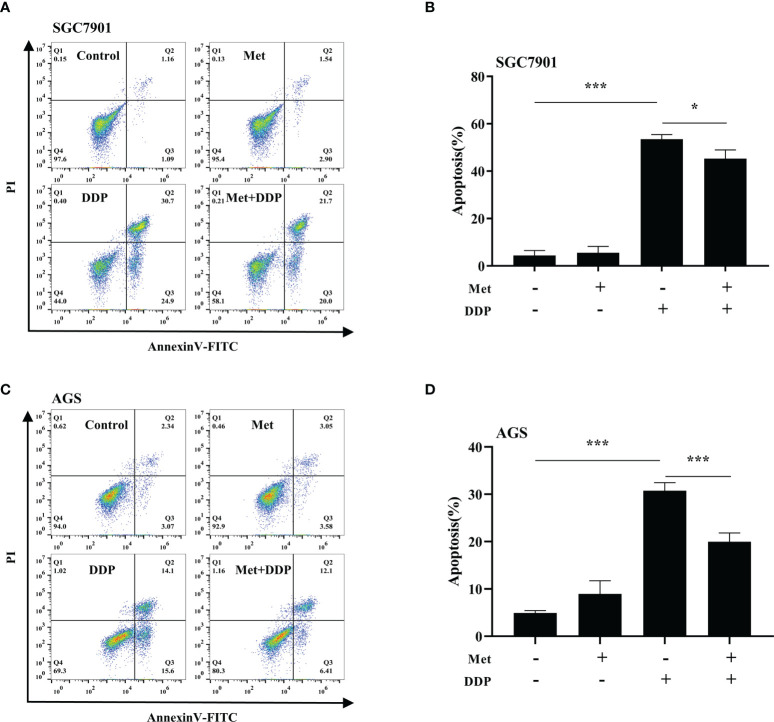
Metformin alleviates cisplatin-induced apoptosis in GC cells. **(A, B)** SGC-7901 cells were treated with metformin (10 mM), DDP (5 μg/mL), or the combination of metformin (10 mM) and DDP (5 μg/mL) for 24 h. **(C, D)** AGS cells were incubated with metformin (10 mM), DDP (10 μg/mL), or combination of metformin (10 mM) and DDP (10 μg/mL) for 24 h. Cell apoptosis was analyzed by Annexin V/PI staining. Bar diagram represents percentage of apoptotic cells. Data were presented as the means ± SD. The experiments were repeated three times. *p<0.05, ***p<0.001.

The effect of metformin on sensitivity of SGC-7901 and AGS cells to cisplatin was examined. The proliferation of SGC-7901 and AGS cells was inhibited in dose-dependent manner at 24 h or 48 h after cisplatin (**Figures 2A, B**). To evaluate the effect of metformin on the sensitivity of GC cells to cisplatin, both SGC-7901 and AGS cells were pretreated with different concentrations of metformin. CCK-8 results showed that metformin significantly alleviated cisplatin-induced growth inhibition in SGC7901 and AGS cells (**Figures 2C, D**). Furthermore, cell apoptosis was examined in SGC-7901 and AGS cells treated with cisplatin and/or metformin. As shown in **Figures 3A, B**, 5 μg/mL cisplatin induced approximately 53% apoptosis in SGC7901 cells, and pretreatment with 10 mM metformin efficiently inhibited cisplatin-induced apoptosis in SGC7901 cells. The similar outcomes were obtained in AGS cells (**Figures 3C, D**). All these results indicate that metformin may promote the resistance of GC cells against cisplatin.

### Metformin promoted cisplatin resistance *via* PINK1/Parkin-dependent mitophagy in GC

**Figure 4:**
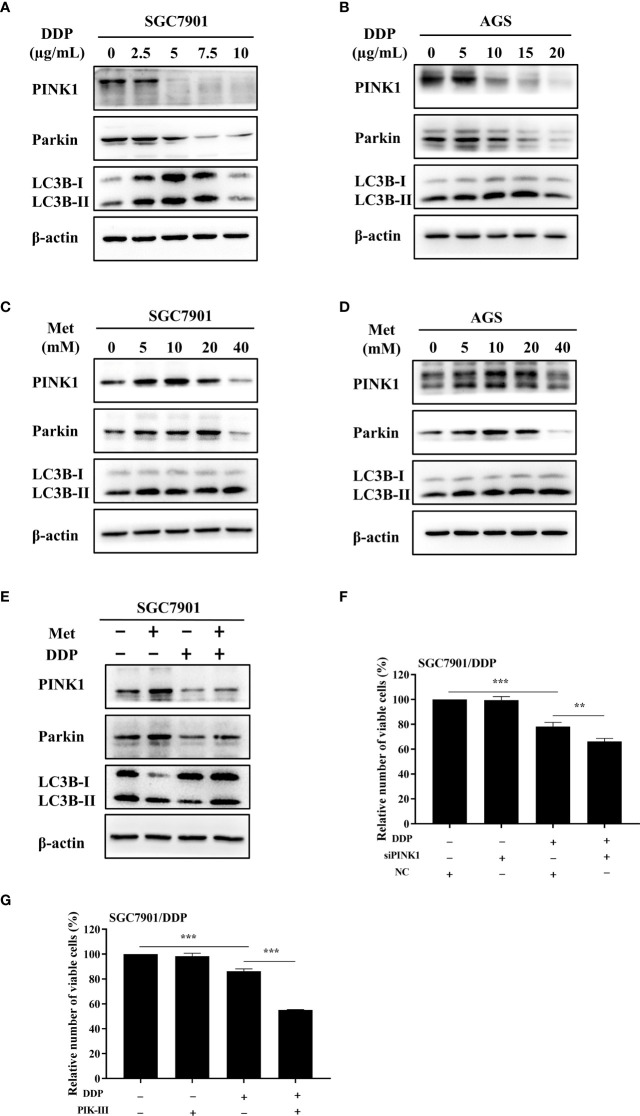
Metformin promoted cisplatin resistance *via* PINK1/Parkin axis in GC cells. **(A, B)** SGC7901 and AGS cells were treated with cisplatin and western blot was performed to determine the expression of PINK1, Parkin, LC3B. **(C, D)** SGC7901 and AGS cells were treated with indicated concentrations of metformin and western blot was performed to determine the expression of PINK1, Parkin, LC3B. **(E)** SGC7901 cells were pre-treated with 10 mM metformin for 4 h, followed by co-treating with 5 µg/mL cisplatin for 24h. The lysates were collected for western blot assay. **(F)** SGC7901/DDP cells were transfected with PINK1 siRNA or scramble siRNA (NC) for 24h and then treated with cisplatin. Cell proliferation was analyzed by CCK8 assay. **(G)** SGC7901/DDP cells were treated with cisplatin in the presence or absence of 5 µM PIK-III for 24 h. Cell proliferation was analyzed by CCK8 assay. Data were presented as the means ± SD. The experiments were repeated three times independently. **p<0.01, ***p<0.001.

As motioned in **Figures 1C~F**, PINK1/Parkin-dependent mitophagy was increased in cisplatin-resistant SGC7901/DDP cells. Thus, the mechanism of PINK1/Parkin-dependent mitophagy in metformin-induced cisplatin resistance was further explored. It was found that the expression of PINK1, Parkin and the ratio of LC3BII/I in both SGC7901 and AGS cells treated with cisplatin was decreased in dose-dependent manner (**Figures 4A, B**; **Figures S3A, B**). Furthermore, the effect of metformin on PINK1/Parkin signaling was examined in two GC cell lines. As shown in **Figures 4C, D** and **Figures S3C, D**, metformin significantly elevated the expression of Parkin, PINK1 and the ratio of LC3BII/I, indicating that metformin might activate PINK1/Parkin signaling. Then, SGC7901 cells were treated with cisplatin and/or metformin. Western blot results showed that metformin attenuated cisplatin-induced downregulation of PINK1, Parkin and the ratio of LC3B-II/I in the SGC-7901 cells (**Figure 4E** and **Figure S3E**). To examine whether the increased expression of PINK1 and ratio of LC3BII/I protect gastric cancer cells from cisplatin, SGC7901/DDP cells were transfected with PINK1 siRNAs to knockdown the expression of PINK1 or treated with PIK-III to down-regulate the ratio of LC3BII/I. PINK1 siRNA#1 showed best knockdown efficiency and was used in following experiments (**Figure S4**). As shown in **Figures 4F, G**, treatment with 5 µM PIK-III for 24 h or PINK1 knockdown alone had no effect on cell viability in SGC7901/DDP cells. However, combination of PIK-III or PINK1 knockdown with cisplatin showed more efficiency in inhibiting cell viability than cisplatin alone, although the inhibitory efficacy was weaker than AMPKα knockdown. These data suggest that cisplatin-resistance in gastric cancer cells is partially related to increased expression of PINK1 and up-regulated ratio of LC3BII/I, and AMPK might affect cisplatin resistance through other pathways.

**Figure 5:**
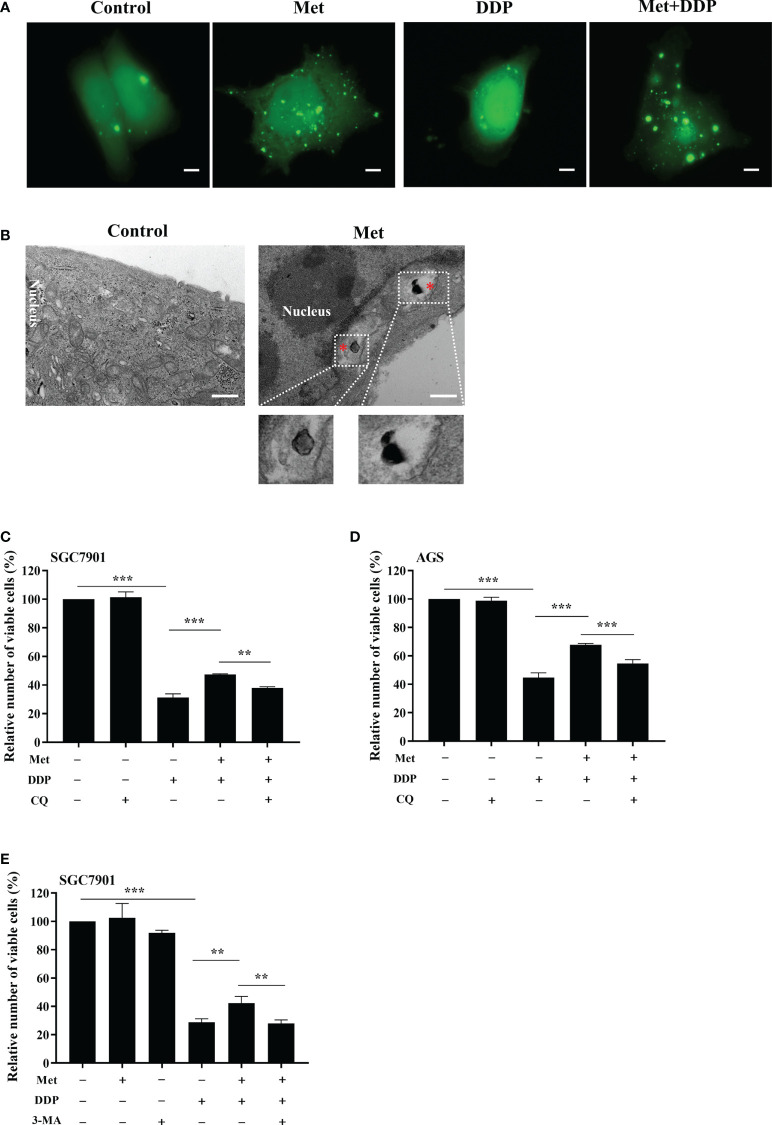
Metformin promoted cisplatin resistance *via* mitophagy in GC cells. **(A)** SGC7901 cells were transfected with pEGFP-LC3B for 24 h. Then cells were treated with indicated drug and analyzed by fluorescence microscopy. Scale bar=5 µm. **(B)** SGC7901 cells were treated with metformin and analyzed by transmission EM. Asterisk represents mitophagy vesicles. Circled images at higher magnification are shown in the below panels. Scale bar=0.5 µm. **(C, D)** SGC7901 and AGS cells were pre-treated with 10 mM metformin followed by co-treating with DDP in the absence or presence of 10 μM CQ for 24 **(h)** Cell viability was analyzed by CCK8 assay. **(E)** SGC7901 cells were pre-treated with 10 mM metformin followed by co-treating with cisplatin in the absence or presence of 5 mM 3-MA for 24 h. Cell proliferation was analyzed by CCK8 assay. Data were presented as the means ± SD. The experiments were repeated three times independently. **p<0.01, ***p<0.001.

To observe the autophagosome formation in live cells, SGC7901 cells were transfected with GFP-LC3B plasmid. The punctate aggregates of GFP-LC3B were decreased in SGC7901 cells treated with cisplatin, which could be reversed by metformin (**Figure 5A**). Moreover, the results of TEM also indicated that autophagic vacuoles were detected in SGC7901 cells treated with metformin (**Figure 5B**). These results suggested that mitophagy was involved in metformin-induced cisplatin resistance. To furthermore confirm our speculation, AGS and SGC-7901 cells were pretreated with 3-methyladenine (3-MA) or Chloroquine (CQ) prior to cisplatin. As shown in **Figures 5C, D**, CQ significantly alleviated the inhibitory effect of metformin on cisplatin-induced growth inhibition in SGC-7901 and AGS cells. Similar outcomes were obtained when SGC7901 cells were pretreated with 3-MA (**Figure 5E**). These data suggest that metformin may promote cisplatin resistance in GC cells *via* PINK1/Parkin dependent mitophagy.

### Metformin stimulated mitochondrial fission and decreased the intracellular ATP level in GC cells

**Figure 6:**
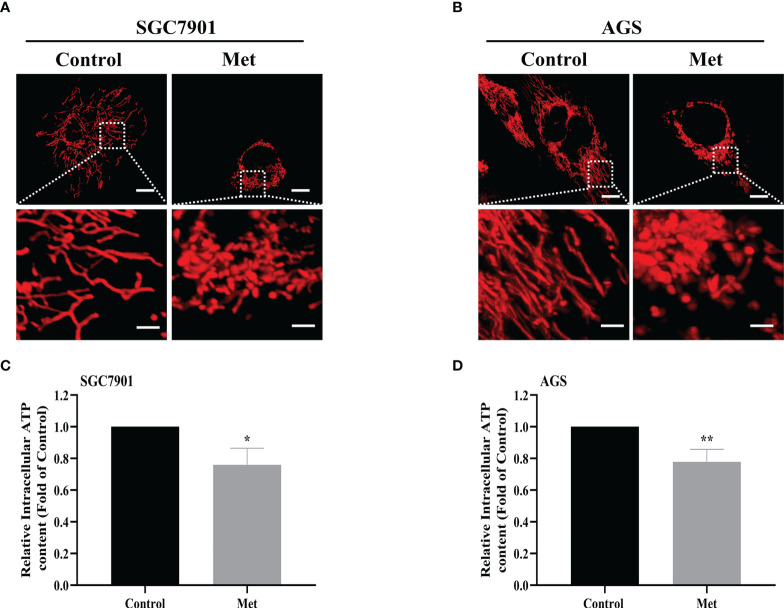
Metformin stimulated mitochondrial fission and decreased the intracellular ATP level in GC cells. **(A, B)** Mitochondrial morphology was shown. SGC7901 and AGS cells were transfected with pDsRed2-Mito and treated with or without 10 mM metformin for 24 h. Scale bar=10 µm. Circled images at higher magnification are shown in the below panels, scale bar=2 µm. **(C, D)** SGC7901 and AGS cells were incubated with 10 mM metformin for 24 h, then ATP levels were detected by ATP assay kit. Data were presented as the means ± SD. The experiments were repeated three times independently. *p<0.05; **p<0.01.

As the increased mitochondrial fission was detected in SGC7901/DDP cells (**Figures 1A, B**), the influence of metformin on mitochondrial dynamics in GC cells was examined. To label mitochondria in live cells, two GC cell lines were transfected with transfected with pDsRed2-Mito plasmid. As shown in **Figures 6A, B**, mitochondria in GC cells appeared as the tubular networks or thread-like structures. In contrast, mitochondria were changed into punctate structures in GC cells treated with metformin, and metformin shortened the average length of mitochondria in GC cells (**Figures S5A, B**). Mitochondria are the major source for ATP production. Therefore, intracellular ATP level was detected in SGC-7901 and AGS cells. As expected, metformin significantly decreased the intracellular ATP levels in two GC cell lines (**Figures 6C, D**). All these results indicated that metformin regulated mitochondrial dynamics, which might contribute to mitophagy and prevent ATP generation.

### Metformin induced mitophagy and cisplatin resistance through AMPK in GC cells

**Figure 7:**
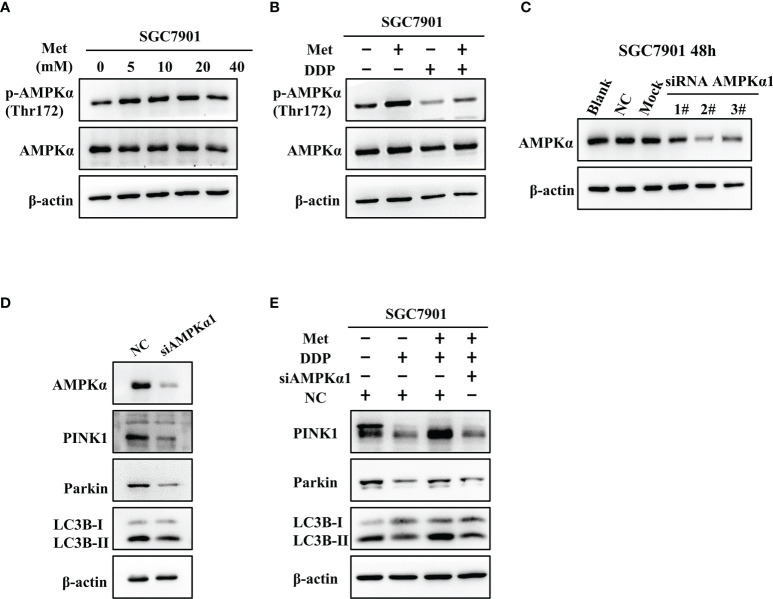
AMPK was involved in the metformin-induced up-regulation of mitophagy-related proteins in GC cells. **(A)** SGC7901 cells were treated with metformin for 24 h. The expression of AMPKα and phos-AMPKα (Thr172) was examined by western blot. **(B)** SGC7901 cells were pre-treated with 10 mM metformin followed by co-treating with 5 µg/mL cisplatin for 24 h. Then, cell lysates were collected for western blot using AMPKα- and phos-AMPKα (Thr172)-specific antibodies. **(C)** Expression of AMPK was determined by western blot in SGC-7901 cells transfected with siRNA-1~3 at 50nM or scramble siRNA (NC). **(D)** SGC7901 cells were transfected with control siRNA or AMPK siRNA#2 and the expression of PINK1, Parkin and LC3B was determined by western blot. **(E)** SGC7901 cells were transfected with scramble siRNA (NC) or AMPK siRNA#2 for 24 h. Then, cells were pre-treated with 10 mM metformin followed by co-treating with 5 µg/mL cisplatin for 24 h. The expression of PINK1, Parkin and LC3B was determined by western blot.

**Figure 8:**
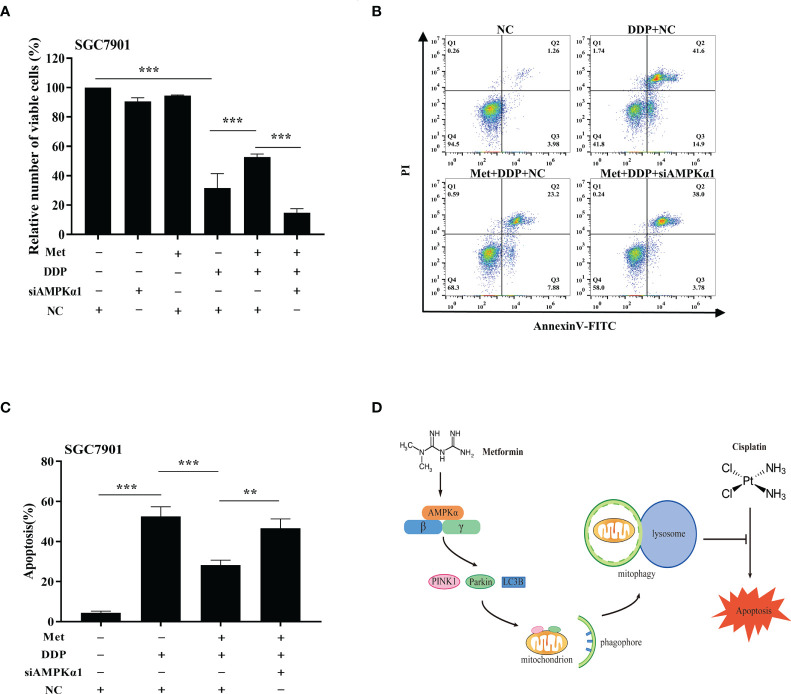
AMPK was involved in the metformin-induced mitophagy in GC cells. **(A)** SGC7901 cells were transfected with AMPK siRNA#2 or scramble siRNA (NC) for 24 h. Then, cells were pre-treated with 10 mM metformin followed by co-treating with 5 µg/mL cisplatin for 24 h. Cell viability was detected by CCK8 kit. **(B, C)** SGC7901 cells were transfected with scramble siRNA (NC) or AMPK siRNA#2 for 24 h. Then, cells were pre-treated with 10 mM metformin followed by co-treating with 5 µg/mL cisplatin for 24 h. Cell apoptosis was analyzed by Annexin V/PI staining. Bar diagram represents percentage of apoptotic cells. Data were presented as the means ± SD. The experiments were repeated three times independently. **p<0.01, ***p<0.001. **(D)** A working model depicting the mechanism of metformin-induced cisplatin resistance in GC cells.

It is well-known AMPK acts as an upstream regulator of PINK1/Parkin dependent mitophagy and mitochondrial homeostasis. Hence, we examined if AMPK participated in metformin-induced cisplatin resistance *via* mitophagy. It was found that metformin up-regulated the expression of phos-AMPKα (Thr172), but not total AMPK expression in SGC7901 cells (**Figure 7A** and **Figure S6A**). However, cisplatin down-regulated the expression of phos-AMPKα (Thr172), which was attenuated by pretreatment with metformin (**Figure 7B** and **Figure S6B**). To examine the role of AMPK in metformin-mediated mitophagy and cisplatin resistance, RNAi of AMPKα1 was conducted in SGC7901 cells using siRNAs. As shown in **Figure 7C** and **Figure S6C**, siRNA #2 showed the best knockdown efficiency on AMPKα1 expression. Thus, siRNA #2 was used for AMPKα1 silencing in the subsequent experiments. Intriguingly, knockdown of AMPKα1 by siRNA down-regulated the expression of PINK1, Parkin and LC3BII/I ratio in SGC7901 cells (**Figure 7D** and **Figure S6D**). In addition, AMPKα1 siRNA alleviated metformin-induced up-regulation of PINK1, Parkin and ratio of LC3BII/I in SGC7901 cells treated with cisplatin (**Figure 7E** and **Figure S6E**). Importantly, metformin mediated suppression on cisplatin-induced growth inhibition and apoptosis was also significantly prevented by AMPKα1 siRNA in SGC7901 cells (**Figures 8A–C**). In conclusion, all these data suggest that AMPK signaling may participate in the metformin-induced mitophagy, and metformin facilitates cisplatin resistance through AMPKα/PINK1/Parkin axis in gastric cancer cells.

## Discussion

Gastric cancer is the third most common cause of cancer death globally. Primary or acquired drug resistance is the major challenge to GC therapy, which inevitably leads to recurrence and poor prognosis in clinic. Previous studies demonstrated that metformin exhibited anti-cancer properties, especially in patients with diabetes (22, 23). Mitophagy acts as a double-edged sword in the chemotherapy for gastric cancer (24, 25). However, the role of metformin-induced mitophagy in cisplatin resistance in GC remains unknown.

Combined application of metformin and cisplatin had shown controversial results in the treatment of various tumors. It has been reported that metformin could promote the anticancer effect of cisplatin in breast cancer (18), NSCLC (26), nasopharyngeal carcinoma (20), liver cancer (27). On the contrary, metformin reduced the anti-proliferative effects of cisplatin through mTOR/AKT signaling pathways in the MKN-45 cells (28). Metformin could also protect OSCC cells from cisplatin toxicity by increasing glycolysis and intracellular NAD(P)H production (29). Additionally, metformin can reduce cisplatin sensitivity in cancer cells through the activation of Akt (30). In this study, we found that metformin alleviated cisplatin-induced growth inhibition in GC cells. Moreover, cisplatin-induced apoptosis was reversed by metformin. These data indicate that metformin promotes the development of cisplatin resistance in GC.

Mitophagy is a mitochondrial quality control system, which promotes tumorigenesis and cell survival by removing abnormal or damaged mitochondria (31). The PINK1/Parkin signaling is a canonical pathway that regulates mitophagy. Previous studies demonstrated that PINK1/Parkin dependent mitophagy can facilitate chemotherapy resistance in ovarian cancer (32), hepatic carcinoma (33, 34), breast adenocarcinoma (35) and lung cancer (36). The regulatory mechanism underlying metformin regulates PINK and Parkin expression and the ratio of LC3BII/I has been explored previously. It was reported that metformin protects against osteoarthritis through PINK1/Parkin-dependent mitophagy by up-regulating SIRT3 expression (37). Metformin alleviated renal oxidative stress and tubulointerstitial fibrosis *via* activating mitophagy through a p-AMPK-PINK1-Parkin pathway (38). In addition, metformin up-regulated the ratio of LC3BII/I and induced autophagy *via* AMPK/mTOR signaling pathway in hepatocellular carcinoma (39). Similar mechanism might also be involved in metformin-induced cisplatin resistance. Here, we showed that mitophagy was enhanced in cisplatin-resistant GC cells. Metformin activated PINK1/Parkin pathway, which resulted in mitophagy and cisplatin resistance in GC cells. Furthermore, pretreatment with mitophagy inhibitors, CQ and 3-MA, effectively attenuated metformin-induced cisplatin resistance in GC cells. AMPK is a key regulator of PINK1/Parkin dependent mitophagy. Previous studies showed that metformin could activate AMPK by facilitating the phosphorylation at Thr172 on AMPK α (40, 41). Consistent with previous reports, up-regulation of phos-AMPKα (Thr172) was observed in GC cells following metformin treatment. Additionally, metformin-induced cisplatin resistance in GC cells could be counteracted by knockdown of AMPKα1 using siRNA. In conclusion, our results indicate that metformin may facilitate cisplatin resistance in GC cells by AMPK-PINK1/Parkin axis-mediated mitophagy.

On the other hand, mitochondrial dynamics is coordinated by balance between fusion and fission (42). Previous studies have suggested that mitochondrial dynamics could influence chemotherapy sensitivity in cancer cells. In our previous studies, it was found that mitochondrial dynamics participate in cisplatin resistance in ovarian cancer, and the level of mitochondrial fusion was higher in cisplatin resistant ovarian cancer cells (43). However, the contrary results were obtained in GC cells, as increased mitochondrial fission was observed in SGC-7901/DDP cells compared with that in SGC-7901 cells. Interestingly, increased mitochondrial fission and lower ATP level were detected in GC cells after metformin treatment. Since mitochondrial fission facilitates the occurrence of mitophagy, our results suggested that metformin-induced mitochondrial dynamics might also contribute to cisplatin resistance in GC cells.

In conclusion, we demonstrate metformin can promote resistance of GC cells to cisplatin through mitochondrial dynamics and AMPK-PINK/Parkin axis-mediated mitophagy (**Figure 8D**). It suggests mitochondrial dynamics as a promising target to overcome cisplatin resistance and improve the chemotherapy efficacy in cancer treatment. Further, our findings warrant caution when considering metformin for treatment of diabetic patients with GC.

## Data availability statement

The datasets presented in this study can be found in online repositories. The names of the repository/repositories and accession number(s) can be found in the article/**Supplementary Material**.

## Author contributions

Y-YX: investigation, methodology, and project administration. J-XX: investigation, methodology, and project administration. X-YW: formal analysis and writing-original draft preparation. TW: formal analysis and writing-original draft preparation. X-HQ: resources. L-PJ: formal analysis. F-FT: visualization. Z-PC: conceptualization and supervision. X-JH: conceptualization, supervision, writing- reviewing and editing, and funding acquisition. All authors contributed to the article and approved the submitted version.

## Funding

This work was supported by National Natural Science Foundation of China (82060177), Major Discipline Academic and Technical Leaders Training Program of Jiangxi Province (20172BCB22028), the Research Fund for Jiangxi Geriatric Clinical Medical Research Centre (2020BCG74003), the Key Research and Development Program of Jiangxi Province (20192BBG70012) and the Key Projects from Department of Education of Jiangxi Province (GJJ218902).

## Conflict of interest

The authors declare that the research was conducted in the absence of any commercial or financial relationships that could be construed as a potential conflict of interest.

## Publisher’s note

All claims expressed in this article are solely those of the authors and do not necessarily represent those of their affiliated organizations, or those of the publisher, the editors and the reviewers. Any product that may be evaluated in this article, or claim that may be made by its manufacturer, is not guaranteed or endorsed by the publisher.
