# Supplementary material for: Metformin-induced AMPK activation promotes cisplatin resistance through PINK1/Parkin dependent mitophagy in gastric cancer
Source: Front Oncol. 2022 Oct 25;12:956190. doi: 10.3389/fonc.2022.956190 (PMC9641368; doi:10.3389/fonc.2022.956190)
Supplement: Supplementary file 1 [file DataSheet_1.pdf]

Supplementary Figure 1

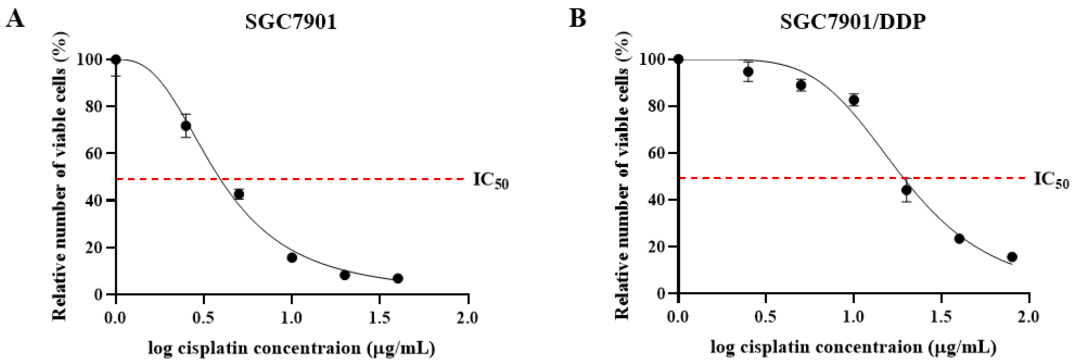

Supplementary Figure 1. Determination of IC50 of cisplatin in SGC-7901 and SGC-7901/DDP cells. (A) The dose-response curve of SGC-7901 cells to cisplatin. **(B)** The dose-response curve of SGC-7901/DDP cells to cisplatin. Cells were treated with cisplatin for 24 h and cell viability was examined by CCK8 assay.

Supplementary Figure 2

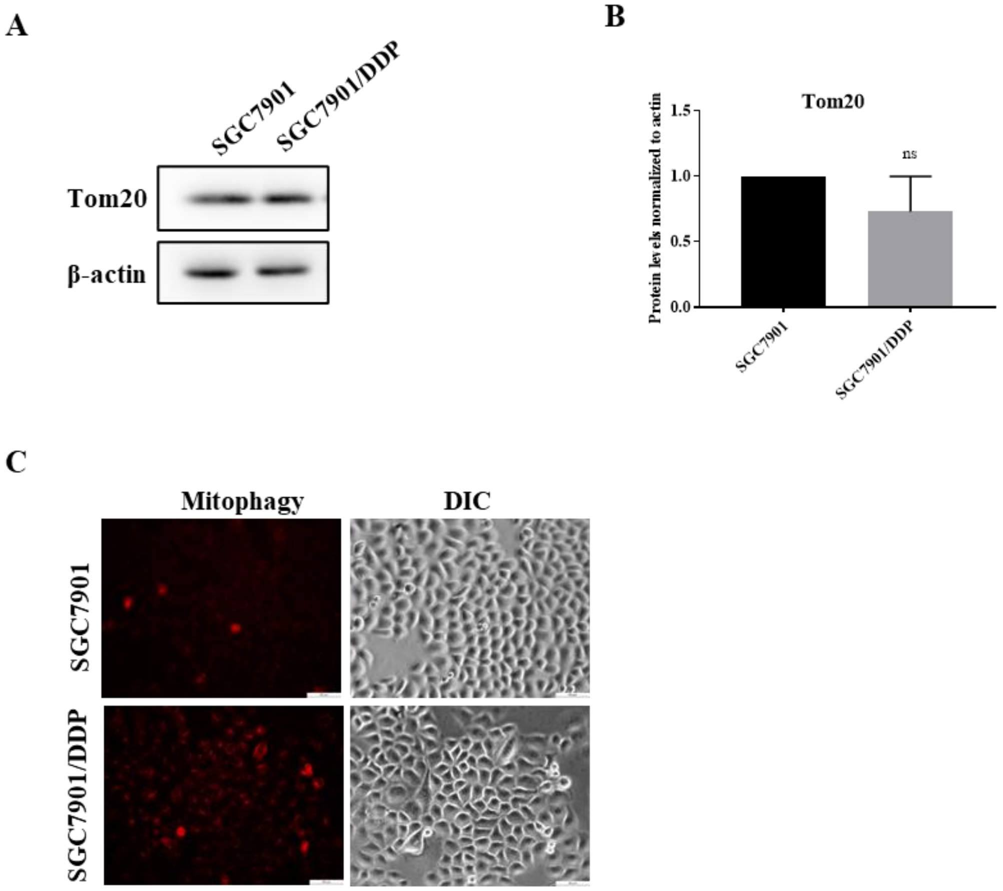

Supplementary Figure 2. Expression of Tom20 and Mtphagy Dye staining in SGC-7901 and SGC-7901/DDP cells. (A) Expression of Tom20 in SGC7901 and SGC7901/DDP cells. Whole cell lysates were collected for western blot assay. **(B)** The graph indicates the quantification of Tom20 expression. The data were normalized to β-actin. ns p>0.05. **(C)** Mitophagy staining in SGC7901 and SGC7901/DDP cells. SGC7901 and SGC7901/DDP cells were stained with Mtphagy Dye and then analyzed by fluorescence microscope. Scale bar=50 μm.

Supplementary Figure 3

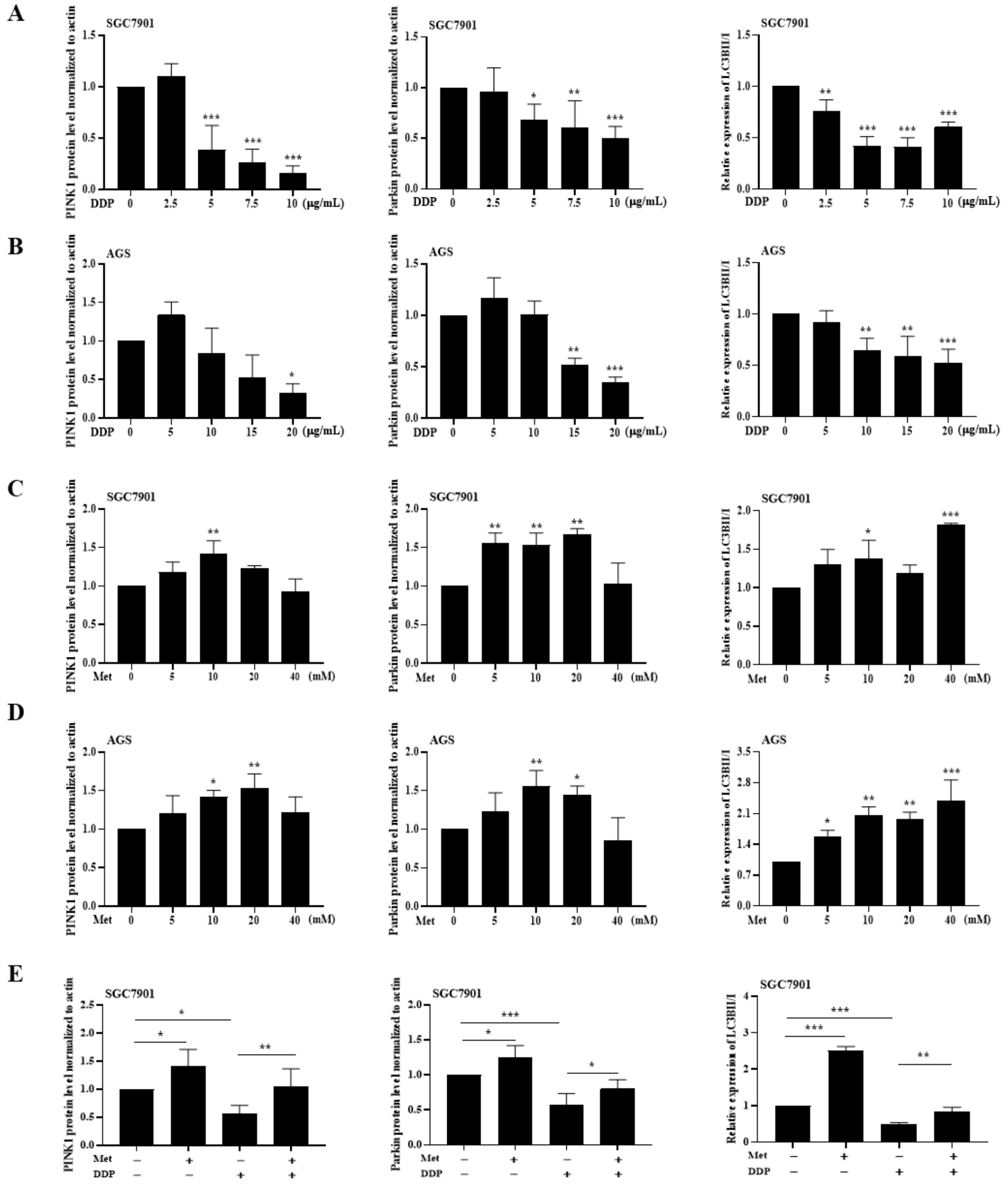

Supplementary Figure 3. Quantitative analysis of western blot results shown in Fig. 4. (A, B) Quantification of Fig. 4A, B. SGC7901 and AGS cells were treated with indicated concentrations cisplatin and western blot was performed to determine the expression of PINK1, Parkin, LC3B. **(C, D)** Quantification of Fig. 4C, D. SGC7901 and AGS cells were treated with indicated concentrations metformin and western blot was performed to determine the expression of PINK1, Parkin, LC3B. **(E)** Quantification of Fig. 4E. SGC7901 cells were pre-treated with 10 mM metformin for 4 h, followed by co-treating with 5 μg/mL cisplatin for 24h. Western blot was performed to determine the expression of PINK1, Parkin, LC3B. β-actin was used as

an equal loading control. Data represents the average of three independent experiments (mean ± SD). The data were normalized to β-actin. *$p<0.05$, **$p<0.01$, ***$p<0.001$.

Supplementary Figure 4

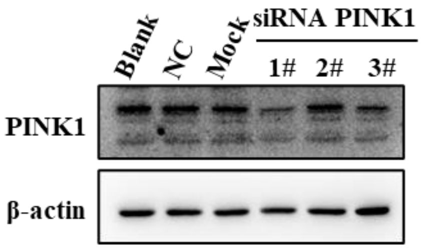

Supplementary Figure 4. Knockdown efficiency of PINK1 siRNAs. SGC7901/DDP cells were transfected with PINK1 siRNAs or scramble RNA. Whole cell lysates were collected for western blot assay.

Supplementary Figure 5

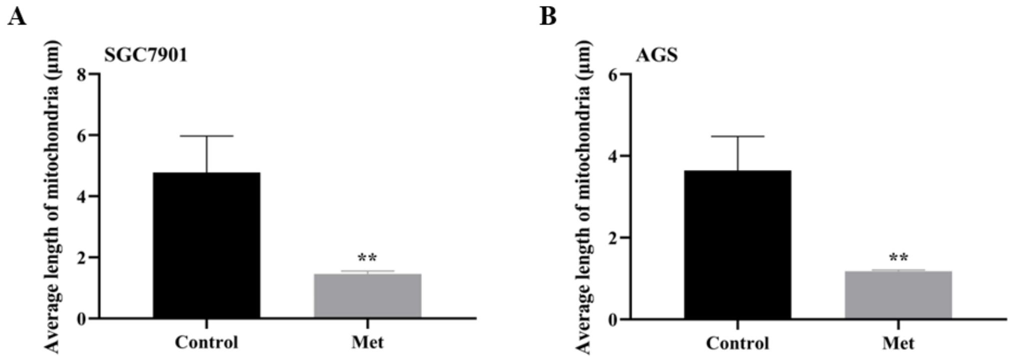

Supplementary Figure 5. Quantitative analysis of confocal imaging results shown in Fig. 6. (A.B) Quantification of Fig. 6A, B. SGC7901 and AGS cells were transfected with pDsRed2-Mito to label mitochondria and treated with or without 10 mM metformin for 24 h. The average length of mitochondria in each group was measured. The data were presented as the mean ± SD. The unit of average length of mitochondria is µm; ***p* < 0.01.

Supplementary Figure 6

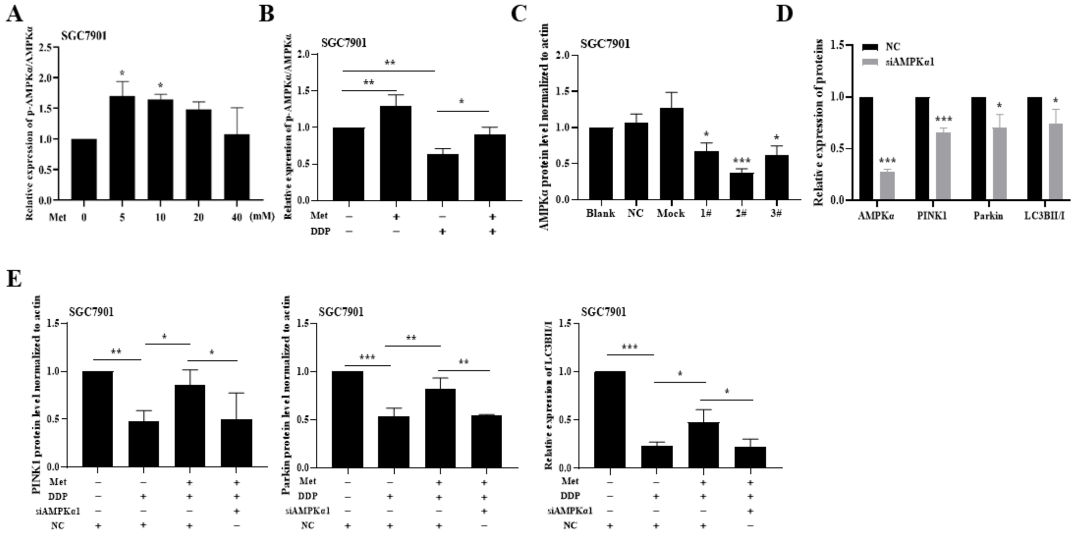

Supplementary Figure 6. Quantitative analysis of western blot results shown in Fig. 7. (A) Quantification of Fig. 7A. SGC7901 cells were treated with indicated concentrations of metformin for 24 h. The expression of AMPKα and phos-AMPKα (Thr172) was examined by western blot. **(B)** Quantification of Fig. 7B. SGC7901 cells were pre-treated with 10 mM metformin followed by co-treating with 5 µg/mL cisplatin for 24 h. Then, cell lysates were collected for western blot analysis using AMPKα- and phos-AMPKα (Thr172)-specific antibodies. **(C)** Quantification of Fig. 7C. Expression of AMPK was determined by western blot in SGC-7901 cells transfected with siRNA-1~3 at 50 nM or scramble siRNA. Cells without transfection were used as controls. **(D)** Quantification of Fig. 7D. SGC7901 cells were transfected with control siRNA or AMPK siRNA#2 and the expression of PINK1, Parkin and LC3B was determined by western blot. **(E)** Quantification of Fig. 7E. SGC7901 cells were transfected with control siRNA or AMPK siRNA#2 for 24 h. Then, cells were pre-treated with 10 mM metformin followed by co-treating with 5 µg/mL cisplatin for 24 h. The expression of PINK1, Parkin and LC3B was determined by western blot. Data were presented as the means ± SD. The experiments were repeated three times independently and the data of one representative experiment was shown. **p*<0.05; ***p*<0.01, ****p*<0.00
